# Omental splenosis causing small bowel obstruction in a pediatric patient: A case report

**DOI:** 10.1016/j.ijscr.2025.111849

**Published:** 2025-08-20

**Authors:** Mohammad Al-Jawad, Hamza Boushi, Alamira Dana Dahhan, Mousa Sifat, Maya Shahoud, Ahmad Ghazal

**Affiliations:** a University of Aleppo, Faculty of Medicine, Aleppo, Syria

**Keywords:** Splenosis, bowel obstruction, case report

## Abstract

**Introduction:**

Splenosis, the autotransplantation of splenic tissue post-trauma/splenectomy, is typically benign and asymptomatic. We report the first reported case of omental splenosis in a pediatric patient causing bowel obstruction via adhesions/internal herniation, highlighting diagnostic challenges.

**Case presentation:**

A 10-year-old post-splenectomy male presented with bowel obstruction. Laparotomy revealed omental splenosis causing adhesional herniation. Histopathology confirmed diagnosis after therapeutic omentectomy/adhesiolysis with full recovery.

**Discussion:**

While splenosis is usually incidental, our case demonstrates its potential for severe complications. Acute presentations may require surgery before definitive imaging, emphasizing the need for clinical suspicion in post-splenectomy patients.

**Conclusion:**

This rare pediatric case illustrates splenosis' potential to cause life-threatening obstruction. Surgeons should consider splenosis in post-splenectomy abdominal emergencies, even when imaging is nonspecific.

## Highlights

• The first reported case of post-splenectomy omental splenosis in a pediatric patient.
• Omental adhesions with ectopic spleen required urgent laparotomy and omentectom.
• Successful outcome highlights need for clinical suspicion in post-splenectomy complications.

## 1 Introduction

Splenosis is a benign condition characterized by the autotransplantation of splenic tissue, typically following trauma or splenectomy that disrupts the splenic capsule [1,2]. This ectopic tissue can implant and grow in various parts of the body through direct implantation or hematogenous spread [1]. First described by Buchbinder and Lipkoff in 1939 [2], splenosis must be distinguished from other splenic anomalies such as accessory spleens and wandering spleens [1], While splenosis can appear in various parts of the body, it is most commonly found in the abdominal and pelvic regions, particularly on the parietal peritoneum, mesentery, omentum, bowel serosa, and diaphragm often mimicking the spread pattern of peritoneal metastases. Less frequently it may appear in atypical locations [2], Most cases are asymptomatic and are detected incidentally on imaging [1] but in rare cases, complications such as bowel obstruction may arise due to the implants effect of the transplanted nodules, intramural splenic growth, mesentery scarring or adhesion formation [3,4].

This case report describes an exceptionally rare occurrence (the first reported case of post-splenectomy omental splenosis in a pediatric patient causing bowel obstruction via adhesions/internal herniation), where omental splenic implants developed adhesions to the abdominal wall, resulting in internal herniation and subsequent small bowel obstruction. We detail the diagnostic and therapeutic challenges of managing this complex presentation while adhering to the SCARE guidelines for surgical case reporting [5].

## 2 Case presentation

**Fig. 1:**
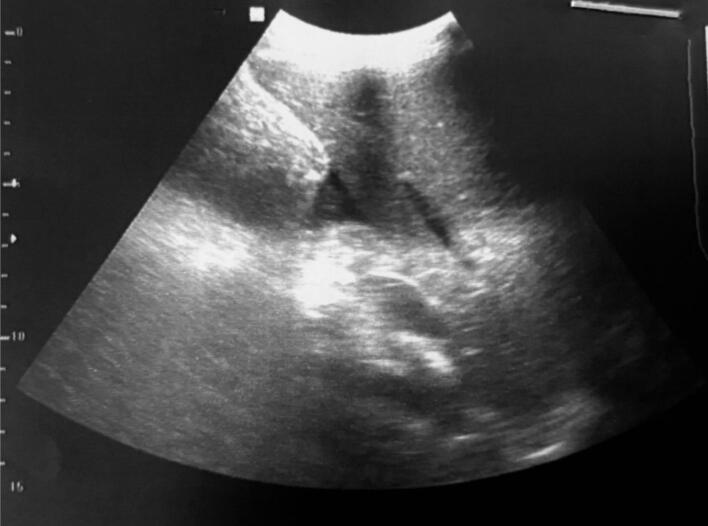
Preoperative ultrasound showed minimal free fluid.

A 10-year-old male presented with a three-day history of progressively worsening abdominal pain, absolute constipation, and bilious vomiting. He had a history of abdominal trauma and splenectomy 10 months prior. Initial laboratory findings included leukocytosis (WBC 14.7 × 10^3^/μL) with neutrophilia (85.1 %), marked thrombocytosis (PLT 1178 × 10^3^/μL), and mild anemia (Hgb 10.3 g/dL). Preoperative abdominal ultrasound revealed minimal free peritoneal fluid without other significant abnormalities Fig. 1**.**

Exploratory laparotomy demonstrated dense adhesions between the greater omentum and anterior abdominal wall, along with approximately 100 mL of serous peritoneal fluid, correlating with ultrasound findings. Intraoperatively, an internal hernia was identified, caused by small bowel loops entrapped within adhesions to omental tissue containing heterotopic splenic implants.

**Fig. 2:**
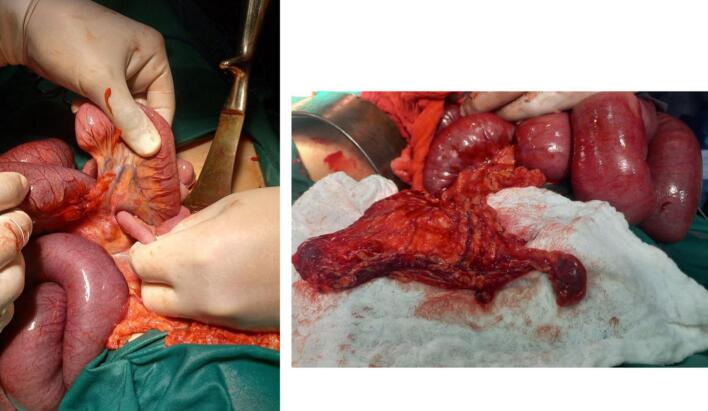
Intraoperative view showing meticulous adhesiolysis followed by reduction of the herniated bowel. An omentectomy was performed with removal of ectopic splenic tissue.

**Fig. 3:**
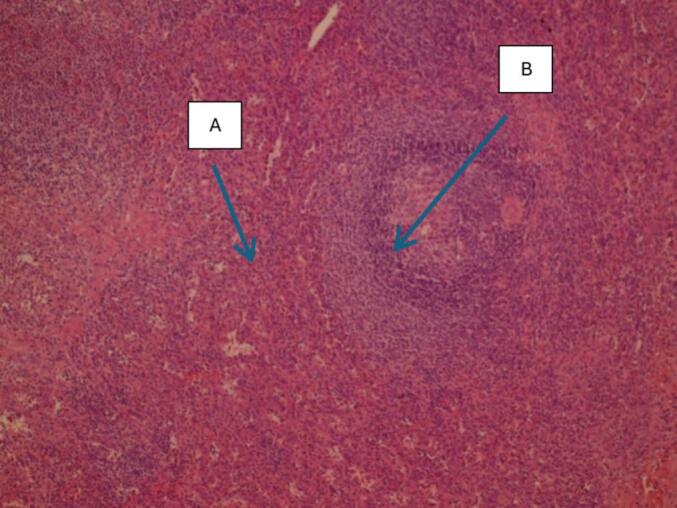
Histopathology (H&E stain×100 magnification) showing: (A): Low-power view showing overall splenic architecture. (B): Higher-power view of a white pulp follicle with germinal center.

The presence of free peritoneal fluid, though nonspecific, raised suspicion for bowel compromise, prompting timely surgical intervention. The fluid was determined to be reactive in nature, secondary to the obstructive process. Surgical management included meticulous adhesiolysis, reduction of the herniated bowel, and omentectomy with removal of ectopic splenic tissue Fig. 2.The resected omental implants was sent for histopathological examination, which confirmed splenosis, showing characteristic splenic tissue architecture with both red and white pulp components Fig. 3**.**

Postoperatively, the patient showed rapid clinical improvement with normalization of laboratory parameters. This case highlights splenosis as a rare but important cause of small bowel obstruction in post-splenectomy patients, necessitating a high index of suspicion for timely diagnosis and intervention.

## 3 Discussion

Ectopic splenic tissue is categorized into two distinct types: congenital and acquired. Congenital ectopic splenic tissue, known as accessory spleens, arises due to the failure of fusion during embryologic splenic development. These accessory spleens are present in approximately 20 % of the population and exhibit normal histologic architecture and functional capacity. They are typically located in the splenic hilum, gastrosplenic ligament, splenorenal ligament, or splenocolic ligament, receiving vascular supply via branches of the splenic artery [6].

Splenosis, resulting from splenic rupture—often associated with diaphragmatic injury—typically presents as small, scattered nodules due to their lack of an independent vascular supply. While these ectopic splenic implants are usually asymptomatic, they may cause recurrent abdominal pain or small bowel obstruction due to adhesive bands formed by the implants. Clinically, patients with splenosis frequently require surgical intervention for complications such as intestinal obstruction or suspected appendicitis. Numerous documented cases of splenosis have been reported in the literature [7].

In our case, the patient developed symptomatic splenosis following traumatic splenectomy, with ectopic splenic tissue forming adhesive bands that caused small bowel obstruction—a known but uncommon complication. Unlike typical asymptomatic scattered nodules, his presentation required surgical intervention for acute intestinal obstruction, highlighting that splenosis, though usually benign, can lead to significant morbidity in rare cases. This aligns with literature reports of splenosis causing mechanical complications rather than just mimicking other abdominal pathologies.

Splenosis is often incidentally detected on imaging (ultrasound, CT, or MRI), though these modalities alone cannot definitively confirm the diagnosis. Contrast-enhanced CT may suggest splenosis in patients with prior splenic trauma or splenectomy, while MRI with iron oxide contrast can help identify ectopic splenic tissue due to its reticuloendothelial uptake. The gold standard remains nuclear scintigraphy using Technetium-99 m-labeled heat-damaged RBCs, which is highly sensitive and specific compared to sulfur colloid scans. Once diagnosed, asymptomatic cases require no treatment, but symptomatic patients may need surgical intervention [8].

In our case, splenosis was diagnosed intraoperatively during emergency exploration for bowel obstruction, unlike the standard approach using Tc-99 m scintigraphy or contrast-enhanced imaging. While imaging typically guides diagnosis, acute presentations may require direct surgical intervention, with histopathology confirming splenosis post-resection. This highlights that symptomatic, complicated splenosis may bypass routine diagnostic protocols.

In our patient, we opted for surgical intervention due to the patient's acute small bowel obstruction caused by adhesional entrapment from splenosis implants, which posed a risk of bowel ischemia. His symptomatic presentation (bilious vomiting, absolute constipation) and imaging findings (free fluid suggesting possible compromise) necessitated emergency exploration. The confirmed presence of obstructive heterotopic splenic tissue mandated adhesiolysis and omentectomy as both diagnostic and therapeutic measures.

The patient's family expressed relief and satisfaction following the resolution of the obstructive symptoms and the uneventful recovery.

## 4 Conclusion

The first reported case of post-splenectomy omental splenosis in a pediatric patient causing bowel obstruction via adhesions/internal herniation. The case underscores that while splenosis typically remains asymptomatic, it can produce serious mechanical complications requiring urgent surgical intervention in rare instances. Our experience reinforces the need to include splenosis in the differential diagnosis of abdominal emergencies in previously splenectomized patients, particularly when classic imaging findings may be absent in acute presentations.

## Informed consent

Unnecessary, information taken from the patient's file.

## Consent for publication

All authors provide consent for publication.

## Ethical approval

Not applicable.

## Guarantor

Ahmad Ghazal

## Research registration number

Our research study does not involve human subjects.

## Provenance and peer review

Not commissioned, externally peer-reviewed.

## Sources of funding

This research did not receive any funding from any external sources. All activities related to this research were conducted without financial support.

## Declaration of competing interest

The authors declare that they have no competing interests.
